# West Nile Virus Epidemics in North America Are Driven by Shifts in Mosquito Feeding Behavior

**DOI:** 10.1371/journal.pbio.0040082

**Published:** 2006-02-28

**Authors:** A. Marm Kilpatrick, Laura D Kramer, Matthew J Jones, Peter P Marra, Peter Daszak

**Affiliations:** **1**The Consortium for Conservation Medicine, New York, New York, United States of America; **2**Wadsworth Center, New York State Department of Health, Albany, New York, United States of America; **3**Smithsonian Environmental Research Center, Edgewater, Maryland, United States of America; Institute for Ecosystem StudiesUnited States of America

## Abstract

West Nile virus (WNV) has caused repeated large-scale human epidemics in North America since it was first detected in 1999 and is now the dominant vector-borne disease in this continent. Understanding the factors that determine the intensity of the spillover of this zoonotic pathogen from birds to humans (via mosquitoes) is a prerequisite for predicting and preventing human epidemics. We integrated mosquito feeding behavior with data on the population dynamics and WNV epidemiology of mosquitoes, birds, and humans. We show that *Culex pipiens,* the dominant enzootic (bird-to-bird) and bridge (bird-to-human) vector of WNV in urbanized areas in the northeast and north-central United States, shifted its feeding preferences from birds to humans by 7-fold during late summer and early fall, coinciding with the dispersal of its preferred host (American robins, Turdus migratorius) and the rise in human WNV infections. We also show that feeding shifts in *Cx. tarsalis* amplify human WNV epidemics in Colorado and California and occur during periods of robin dispersal and migration. Our results provide a direct explanation for the timing and intensity of human WNV epidemics. Shifts in feeding from competent avian hosts early in an epidemic to incompetent humans after mosquito infection prevalences are high result in synergistic effects that greatly amplify the number of human infections of this and other pathogens. Our results underscore the dramatic effects of vector behavior in driving the transmission of zoonotic pathogens to humans.

## Abstract 2

A shift in the feeding behavior of mosquitoes from birds to mammals in late summer amplifies the number of human infections of West Nile virus in the northeast and north-central US.

## Introduction

Approximately three quarters of human emerging infectious diseases are caused by zoonotic pathogens [ 1]. For many of these diseases, nonhuman animals are the primary host and human infections are incidental and often dead-end in nature [ 1]. Nonetheless, these pathogens can have severe consequences for human health, due to high mortality rates, high incidence rates, or both [ 1– 4]. The factors that drive the spillover of these pathogens from other animals to humans determine the intensity of human epidemics [ 5]. For directly transmitted zoonotic pathogens, transmission to humans increases with the contact rate between infected animals and susceptible humans, whereas for vector-borne diseases, the feeding behavior and feeding preferences of the vector(s) play a key role in determining the force of infection [ 6]. Therefore, a critical step in the control of epidemics of zoonotic vector-borne pathogens is determining the feeding preferences of key vectors and how they change over space and time [ 5].

West Nile virus (WNV) is a zoonotic pathogen that is maintained in an enzootic cycle primarily through transmission between viremic birds and ornithophilic (bird-biting) mosquitoes [ 7]. However, during late summer and early fall, WNV also infects humans and other mammals and has caused repeated large-scale human epidemics in North America since it was first detected in New York City in 1999 [ 8]. It has caused over 20,000 reported cases, 770 deaths, and an estimated 215,000 illnesses during the past 7 y and is now the dominant vector-borne disease in North America [ 9– 11]. During the past 4 y, there were epidemics in North America of approximately 2,000 to 10,000 cases each, far larger than European epidemics, which have never exceeded 400 cases and occur infrequently [ 12].

Three hypotheses have been put forth to explain the increased intensity of epidemics in North America. First, the strain of WNV introduced in North America has been shown to be more virulent to American crows *(Corvus brachyrhynchos)* than one strain circulating in the Old World [ 13]. Second, North American birds had no previous exposure to WNV and the lack of acquired and evolved immunity may have increased the intensity of epidemics [ 14]. Third, North American Culex pipiens mosquitoes, the dominant vector of WNV in the northeast and north-central United States, appear to be hybrids of the bird-biting “pipiens” form and human-feeding “molestus” form of Old World *Cx. pipiens* [ 15]. These three hypotheses are neither mutually exclusive nor exhaustive.

We tested a fourth hypothesis, that epidemics are driven by a shift in mosquito feeding behavior from birds to mammals, as has been demonstrated for other mosquitoes in North America [ 16– 18] but not for *Cx. pipiens* in the New or Old World [ 19]. The most competent vertebrate hosts for WNV appear to be birds [ 20], whereas most mammals, including humans, are poorly competent hosts [ 21, 22]. If mosquitoes feed primarily on birds in the summer and then switch to humans in the fall, this may amplify both the intensity of the summer epidemic in mosquitoes and birds and later transmission of WNV to humans. To test this hypothesis and to understand the factors driving North American human WNV epidemics, we integrated data on the feeding behavior, population dynamics, and epidemiology of mosquitoes, birds, and humans in three regions across the United States.

## Results

### PCR and DNA Sequencing of Mosquito Blood Meals

The probability that *Cx. pipiens* fed on humans increased over the mosquito season ( Figure 1A; logistic regression of individual blood meals on Julian date: intercept = −7.3, slope = 0.025; *n* = 148 blood meals; *p* = 0.004). The probability that *Cx. pipiens* fed on mammals also increased ( Figure 1A; intercept = −6.8, slope = 0.025; *n* = 148 blood meals; *p* < 0.001). However, the probability of feeding on nonhuman mammals did not increase significantly (intercept = −6.5, slope = 0.017; *n* = 148 blood meals; *p* = 0.14), suggesting that the increase in mosquitoes' preference for humans was responsible for the apparent shift to mammals. As would be expected, the probability of feeding on avian hosts declined over the same period (intercept = 11.9, slope = −0.046; *n* = 148; *p* < 0.001). Using the fitted logistic equations, the probability of *Cx. pipiens* feeding on humans and mammals increased from 0.040 and 0.064 in mid-June to 0.28 and 0.39 in mid-September, which roughly match the monthly groupings of the data ( Figure 1A). These are 7-fold and 6-fold increases, respectively.

**Figure 1:**
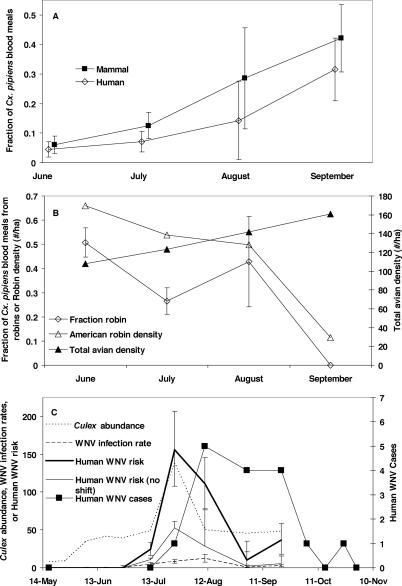
Cx. pipiens Feeding Patterns, Avian Population Dynamics, and West Nile Virus Epidemiology (A) Fraction of 148 *Cx. pipiens* blood meals (±1 SE) from humans and mammals (including humans) identified by PCR and DNA sequencing. (B) Density (birds per hectare) of American robins, density of all birds, and the fraction (±1 SE) of mosquito feedings on robins. (C) Abundance of *Culex* mosquitoes per trap-night, *Culex* WNV infection rate (1,000 * infection prevalence, ±1 SE), estimated human WNV infection risk (±1 SE), calculated as the product of mosquito abundance, WNV infection rate, and the time-varying probability of feeding on humans (Human risk) or the June probability, 0.04 (Human risk – no shift), and the number of human WNV cases in Maryland in 2004.

### Changes in Mosquitoes' Preferred Host

In early summer (May and June), 51% of *Cx. pipiens*' blood meals came from a widespread, competent avian host, the American robin *(Turdus migratorius)* [ 20, 23], despite the fact that this species made up only 4.5% of the avian community ( Figure 1B) (2% to 7% at each site). The abundance of robins in urbanized areas declined over the next 4 mo ( Figure 1B; relative abundance of American robins = 3.25 − 0.0116 * Julian date; *n* = 24; *R* ^2^ = 30.2%; *p* = 0.004), as robins dispersed after breeding. As the availability of robins declined, the probability they were fed upon by *Cx. pipiens* also declined markedly ( Figure 1B; logistic regression of individual blood meals on Julian date: intercept = 6.5, slope = −0.037; *n* = 148; *p* < 0.001), which coincided with the rise of humans as an important source of *Cx. pipiens* blood meals ( Figure 1A). In contrast, the total abundance of birds, which was dominated by house sparrows, Passer domesticus (42% to 67% of total abundance), increased over this period as a result of reproduction ( Figure 1B). Thus, the shift in *Cx. pipiens* feeding from birds to humans is not a result of decreasing avian abundance but is more likely the result of the decline in the abundance of robins, their preferred host.

### Predicted and Actual Human WNV Infections

Our model-predicted risk of the transmission of WNV to humans by *Cx. pipiens* peaked from late July to mid-August, declined in late August, and increased slightly at the end of September ( Figure 1C) before a period of cold weather greatly decreased mosquito activity in early October. Human WNV infections in the surrounding area showed a strikingly similar pattern ( Figure 1C), with a steep rise in cases occurring approximately 14 d after the peak in predicted risk, in agreement with the 3- to 14-d period between infection and the onset of illness in humans [ 24] and the approximately 7-d delay between becoming infected and becoming infectious when a mosquito is initially infected with WNV [ 25]. The correlation of our predicted risk and human cases 14 d later was highly significant ( *r* = 0.80; *n* = 8 biweekly accumulations from mid-June through October; *p* = 0.009). Finally, our data-driven model suggests that had mosquitoes continued to feed on humans with the same probability that they did in June, human epidemics would have occurred at much lower intensity and there would have been few, if any, infections after mid-September ( Figure 1C: Human infection risk [no shift]).

### Feeding Shifts and WNV Epidemics in Other Parts of North America

Previously documented shifts from birds to mammals in *Cx. tarsalis* in California [ 18] and Colorado [ 17] also intensify WNV epidemics in these states ( Figure 2). The fraction of feedings from mammals in peak WNV months (July through September) in California and Colorado were 4.1- and 1.7-fold higher, respectively, than those in June, suggesting that feeding shifts substantially increased the number of human WNV cases in these regions. The July and August peaks in human WNV cases in Colorado and California follow the peak in the abundance of WNV-infected mosquitoes that occur in June and July, respectively [ 26, 27], and are amplified and extended by the shift in *Cx. tarsalis* feeding from birds to mammals. These feeding shifts occur during the late summer periods of postbreeding dispersal and migration of robins in these regions ( Figure 2). In support of the link between feeding patterns and the dispersal and decline of passerine migrants, the fraction of feedings of *Cx. tarsalis* from passerines (many of which are competent for WNV [ 20, 28]) decreased from 40.5 ± 1.9% of feedings in May through June to 20.0 ± 1.0% in July through September in Colorado (correlation with time: *r* = −0.90; *n* = 5 mo; *p* = 0.037; data from [ 17]).

**Figure 2:**
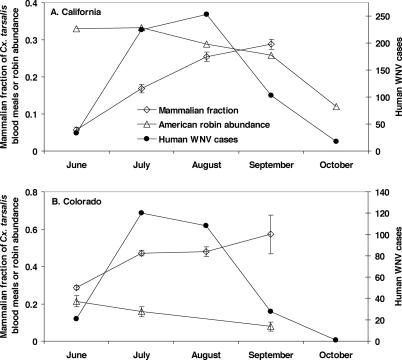
Cx. tarsalis Feeding Patterns, Avian Population Dynamics, and West Nile Virus Epidemiology Fraction of *Cx. tarsalis* feedings from mammals (±1 SE), abundances of American robins [birds/survey in (A), birds/hectare ±1 SD in (B)], and the number of human WNV cases in 2004 in California (A) and Colorado (B).

## Discussion

Our data from the mid-Atlantic demonstrate a late-summer shift in the feeding behavior of *Cx. pipiens* from their preferred avian host, American robins, to humans. This shift offers an explanation for the late-summer timing and increased intensity of human WNV epidemics in the northeast and north-central parts of North America [ 11, 29], where this species is the dominant enzootic vector and bridge vector [ 30]. We have also shown that WNV epidemics in the central and western parts of the United States are intensified by feeding shifts that escalate as robins and other WNV-competent avian hosts disperse and migrate following breeding. Finally, an important WNV vector in the southeast United States, *Cx. nigripalpus* [ 31], also shifts from feeding primarily on birds in spring to an increasing fraction from mammals in later summer, although the cause for this shift is unknown [ 16]. Taken together, these data suggest that mosquito feeding shifts appear to be a continent-wide phenomenon that amplifies the transmission of WNV from birds to humans and other mammals.

Feeding shifts have two synergistic effects on the intensity of WNV transmission to humans. First, we have shown that the increase over time in the probability of *Cx. pipiens* feeding on humans results in a greater number of human WNV infections than if the mosquitoes fed on humans with the same probability as in early summer ( Figure 1C). Second, feeding primarily on WNV-competent avian hosts during the amplification period of the epidemiological cycle maximizes the intensity of the epidemic in mosquitoes. If mosquitoes fed on humans in early summer with the same probability as that seen in late summer (approximately 0.30), the peak and mean prevalence of WNV in mosquitoes would be substantially reduced [ 32, 33], due to the large number of wasted feedings on humans, which, like immune birds, are dead-end hosts [ 21]. In contrast, holding the probability of mosquitoes feeding on humans at its lowest level, 0.04 (as we modeled in Figure 1C), has only a minor effect on WNV amplification. This is because mosquito WNV prevalences are already beginning to decline (possibly as a result of increased acquired immunity in juvenile birds) when mammals begin to make up an important fraction of the blood meals. Thus, the shift in feeding from competent hosts early in the season to humans later leads first to greater amplification of the virus as transmission intensifies between birds and mosquitoes and subsequently to an even greater number of human WNV infections.

Feeding shifts from birds to humans offer a broader and more direct explanation for the intensity of North American WNV epidemics compared to those in Europe than those previously proposed: hybridization of the “pipiens” and “molestus” forms of *Cx. pipiens* [ 15], the novelty of WNV for North American birds [ 14], and the virulence of the introduced strain compared to an Old World isolate [ 13]. First, although it is possible that the feeding shift we observed in *Cx. pipiens* could be the result of increasing hybridization between the molestus and pipiens forms of *Cx. pipiens* [ 15], this has yet to be demonstrated, and a shift due to robin dispersal is a more parsimonious explanation. In any case, increasing hybridization would offer an explanation only in areas where *Cx. pipiens* is the dominant vector. In addition, a temporally invariant increase in feeding on humans by hybrid *Cx. pipiens* as proposed [ 15, 19] would have counteracting effects on mosquito and human WNV infections, as we have shown. Second, WNV epizootics occur in birds after the production of large numbers of naïve and susceptible offspring, so the acquired immunity of adults may play only a limited role in subsequent transmission. Third, the introduced strain of WNV appears to be more virulent compared to an Old World strain in American crows *(Corvus brachyrhynchos)* [ 13], but not in more important amplification hosts such as house sparrows *(Passer domesticus)* [ 34, 35]. Finally, high vector competence of *Cx. tarsalis* [ 36] and increases in the abundance or WNV infection prevalence of more mammalophilic mosquito species may also contribute to WNV transmission to humans. However, the latter appears to be of limited importance in the northeast United States where the enzootic vectors appear to be responsible for more than 80% of human WNV infections [ 30].

In sum, although many factors influence WNV transmission to humans, our results suggest that feeding shifts occur across North America and offer a direct explanation for the greater number of human WNV infections compared to the Old World. More broadly, our findings show that shifts in mosquito feeding behavior are a geographically widespread phenomenon that intensifies epidemics of several avian zoonotic viruses including WNV, Western equine encephalitis virus, and St. Louis encephalitis virus [ 16] *,* and may affect other zoonotic vector-borne pathogens. Studies aimed at determining the factors that influence pathogen transmission to humans are critical to reducing the impact of these and other devastating diseases.

## Materials and Methods

We collected field data at six sites in Maryland and Washington, D. C., from May through September 2004. The sites included three urban areas, the National Mall in Washington, D. C., northeast of the Watergate Hotel in Foggy Bottom, D. C., and west of Camden Yards in Baltimore, Maryland; two residential areas, Takoma Park, Maryland, and Bethesda, Maryland; and one park within an urban setting, Fort Dupont Park, D. C. Each site was approximately 1 km in diameter, with a relatively homogeneous percent forest cover and land use in the site and surrounding area. The sites span a distance of greater than 40 miles and are separated by 3 to 25 miles.

We estimated the abundance of host-seeking *Culex* mosquitoes at each site using eight CDC light traps, baited with dry ice (CO _2_). We ran these traps for two nights every 2 wk from May to September at each site and averaged abundance across the sites. To determine the species composition of *Culex* mosquitoes at our sites, which are difficult to identify morphologically [ 37], we used PCR on the internal spacer gene [ 38] to identify all engorged *Culex* mosquitoes to species and an additional subset of 40 *Culex* mosquitoes at each site. We found that more than 90% of the *Culex* mosquitoes identified at each of the six sites were *Cx. pipiens*.

We collected engorged mosquitoes from each site using CDC light and gravid traps and by collecting mosquitoes resting on vegetation with a large backpack-mounted aspirator. Blood meals were identified as avian, mammalian, or other following extraction of DNA from the engorged mosquitoes and PCR amplification of the cytochrome b gene as described and verified [ 39]. Hosts were identified to the species level by nucleotide sequencing of the amplified product using an ABI 3700 DNA Analyzer (Applied Biosystems, Foster City, California, United States) with avian or mammalian forward and reverse primers [ 39]. Data were analyzed using the DNAStar software package (Madison, Wisconsin, United States) and sequence analysis was conducted using Blastn via the Internet. Only engorged mosquitoes identified as *Cx. pipiens* were used in the feeding analysis. We obtained PCR product for 165 blood meals and DNA sequences for 148 of these.

Mosquitoes were tested for WNV RNA using real-time RT-PCR [ 40] in groups (pools) of 20 to 50 individuals. The WNV infection prevalence was estimated using maximum likelihood techniques [ 41] and expressed as the WNV infection rate = 1,000 × mosquito WNV infection prevalence.

We modeled the risk of the transmission of WNV to humans by *Cx. pipiens* as the product of *Culex* mosquito abundance (more than 90% of which were *Cx. pipiens*), *Culex* WNV infection prevalence, and probability of feeding on humans [ 30]. We calculated the variance of predicted risk using a Taylor series expansion approximation:



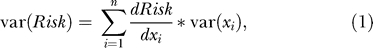



where the *x _i_* parameters represent the *n* = 3 parameters in the risk model. We compared predicted WNV risk to the number of human cases in Maryland [ 42, 43]. We corrected the date of reporting by obtaining the date of the onset of illness for each case (unpublished data, A. Bergmann, Maryland Department of Health and Mental Hygiene).

We estimated the abundance of birds using four- to six-point transects, six minutes in duration, performed at each site monthly from May through September and analyzed the data using Program Distance [ 44], which accounts for species' differences in detectability. We estimated the decline of American robin abundance ( Figure 1B) over time using a regression of the relative abundance of robins at each site on the Julian date. The changes in robin and total bird abundance were similar across the six sites.

To examine the temporal overlap between feeding shifts and human WNV epidemics in other parts of North America, we obtained data on temporal variation in feeding on birds and mammals for *Cx. tarsalis* in Colorado [ 17] and California [ 18]. We compared these patterns with data on the date of onset of illness and number of human cases of WNV in Colorado [ 45] and California [ 27] in 2004. Finally, we compared these to periods of postbreeding dispersal and migration for robins in California [ 23, 46] and in four different habitats in Arizona [ 47– 49].

## Abbreviation

WNV: West Nile virus
